# Draft Genome Sequences of Six *Enterococcus faecalis* Strains Isolated from Malaysian Clinical and Environmental Origins

**DOI:** 10.1128/genomeA.00553-17

**Published:** 2017-06-15

**Authors:** Diane Sunira Daniel, Han Ming Gan, Sui Mae Lee, Gary A. Dykes, Sadequr Rahman

**Affiliations:** a School of Science, Monash University, Bandar Sunway, Malaysia; b Tropical Medicine and Biology Platform, Monash University, Bandar Sunway, Malaysia; c School of Public Health, Curtin University, Perth, Western Australia, Australia

## Abstract

*Enterococcus faecalis* is known to cause a variety of nosocomial infections, including urinary tract infections. Antibiotic resistance and virulence properties in this species are of public concern. The draft genome sequences of six *E. faecalis* strains isolated from clinical and environmental sources in Malaysia are presented here.

## GENOME ANNOUNCEMENT

*Enterococcus faecalis* is an opportunistic pathogen that is often recovered from urinary tract infections (UTIs). *E. faecalis* is known to cause infections mainly due to the expression of virulence factors associated with adherence of mucosal and abiotic surfaces (1). The number of complete or draft genome sequences available for *E. faecalis* as of April 2017 is 503, comprising the bulk of enterococcal genome sequences available. However, there is a poor representation of genomic sequences for enterococci from Malaysia with only approximately seven assemblies reported (2). In this study, six *E. faecalis* strains, designated S12, S13, S14, S15, S16, and S17, were sequenced. These six strains were previously isolated from water sources, farm animal feces, and UTI patients in Malaysia and selected on the basis of different pulsed-field gel electrophoresis pulsotypes reported in a previously published experiment (3), and different biofilm and attachment properties (our unpublished data).

Genomic DNA was extracted using a GF-1 bacterial DNA extraction kit (Vivantis, Malaysia), tagmented with Nextera XT (Illumina, USA) according to the manufacturer’s instructions and sequenced on the MiSeq desktop sequencer located at the Monash University Malaysia Genomics Facility (2 × 250-bp run configuration). The raw reads generated were trimmed (quality score limit of 0.05) and assembled *de novo* using CLC Genomics Workbench version 7.0 (CLC bio, Denmark). Genome annotation was performed using the NCBI Prokaryotic Genome Annotation Pipeline and the Rapid Annotations using Subsystems Technology server (4, 5). The identification of acquired antibiotic resistance and virulence genes was performed with the web tools ResFinder and VirulenceFinder, respectively (6, 7). Contigs coding for each antibiotic resistance and virulence gene (represented by contig accession number) were filtered based on 90% identity to the reference sequence and are presented in Table 1.

**TABLE 1:** Summary characteristics of whole-genome assemblies of six *E. faecalis* strains isolated from various sources in Malaysia^a^

	Parameter	Characteristic for strain:					
		S12	S13	S14	S15	S16	S17
Accession no.	NBDR00000000	NBDS00000000	NBDW00000000	NBDV00000000	NBDU00000000	NBDT00000000
Isolation source	River water	Chicken feces	Chicken feces	River water	UTI patient	UTI patient
Mean coverage (×)	53	70	63	27	80	89
G+C content (%)	37.3	37.4	37.3	37.5	37.5	37.6
*N*_50_ (bp)	38,596	57,066	152,272	36,471	83,501	33,430
No. of contigs	140	133	92	158	110	229
Genome size (bp)	3,002,129	2,934,970	3,065,309	2,877,096	2,856,164	2,861,645
No. of CDSs	2,921	2,857	3,271	2,738	2,698	2,685
No. of tRNAs	59	42	55	37	51	49
No. of rRNAs	4	3	3	4	5	7
Contig accession no. for^b^:						
*vanBHRSXWY*	NA	NA	NBDW01000006	NA	NA	NA
*tetM*	NBDR01000040	NA	NA	NA	NA	NA
*gelE*	NBDR01000048	NBDS01000053	NBDW01000028	NBDV01000016	NBDU01000023	NBDT01000088
*efaA*	NBDR01000100	NBDS01000066	NBDW01000002	NBDV01000033	NBDU01000049	NBDT01000053
*ace*	NBDR01000038	NBDS01000079	NBDW01000009	NBDV01000014	NBDU01000054	NBDT01000152
*ebpC*	NBDR01000050	NBDS01000024	NBDW01000009	NBDV01000060	NA	NBDT01000066
*ebpR*	NBDR01000050	NBDS01000024	NBDW01000009	NA	NBDU01000043	NBDT01000066
*eep*	NBDR01000032	NBDS01000004	NBDW01000006	NBDV01000036	NBDU01000015	NBDT01000113

^a^ Abbreviations: UTI, urinary tract infection; CDSs, coding sequences; NA, not available.

^b^ The accession numbers of the reference sequences for each gene are as follows: *vanBHRSXWY*, KC489787; *tetM*, U09422; *gelE*, DQ845100; *efaA*, JF512477; *ace*, HQ003827; *ebpC*, KJ710255; *ebpR*, EF646762; and *eep*, AF152237. Contigs containing the corresponding gene(s) exhibit ≥90% identity to their respective reference sequence.

Genomic statistics—mean coverage, *N*_50_ contig length, number of contigs, assembly size, number of coding sequences, and number of tRNAs and rRNAs for the six assembled genomes are provided in Table 1. An orthologous average nucleotide identity tool based on OrthoANI values revealed a >98% similarity of all six strains to the whole genome of *E. faecalis* ATCC 19433 (PRJNA157741) (8).

Strain S14 harbors the complete gene cassette for vancomycin resistance, corroborating a previous wet lab report that recorded a MIC value of 64 µg/mL (3), whereas strain S12 was found to have a tetracycline resistance gene (*tetM*) with a MIC value of 32 µg/mL (3). In addition, all six strains harbor genes coding for virulence factors—gelatinase production (*gelE*), endocarditis antigen (*efaA*), collagen adhesion (*ace*), and biofilm-associated pili (*eep*)—that are associated with adherence and invasion of the host tissue among enterococcal strains (9). Genes *ebpC* and *ebpR*, which are also biofilm-associated virulence factors, were identified in all strains except S16 and S15, respectively.

### Accession number(s).

The whole genome shotgun project of *E. faecalis* strains S12, S13, S14, S15, S16, and S17 have been deposited in DDBJ/ENA/GenBank under the accession numbers listed in Table 1.
